# *Iditarod*, a *Drosophila* homolog of the Irisin precursor *FNDC5*, is critical for exercise performance and cardiac autophagy

**DOI:** 10.1073/pnas.2220556120

**Published:** 2023-09-18

**Authors:** Tyler Cobb, Irene Hwang, Michael Soukar, Sim Namkoong, Uhn-Soo Cho, Maryam Safdar, Myungjin Kim, Robert J. Wessells, Jun Hee Lee

**Affiliations:** ^a^Department of Physiology, Wayne State University School of Medicine, Detroit, MI 48201; ^b^Department of Molecular & Integrative Physiology, University of Michigan, Ann Arbor, MI 48109; ^c^Department of Biochemistry, College of Natural Sciences, Kangwon National University, Chuncheon, Gangwon 24341, Republic of Korea; ^d^Department of Biological Chemistry, University of Michigan, Ann Arbor, MI 48109

**Keywords:** exercise, Irisin, FNDC5, cardiac, autophagy

## Abstract

Mammalian *FNDC5* encodes a protein precursor of Irisin, which is important for exercise-dependent regulation of whole-body metabolism. In a genetic screen in *Drosophila*, we identified *Iditarod* (*Idit*), which shows substantial protein homology to mouse and human *FNDC5*, as a regulator of autophagy acting downstream of Atg1/Atg13. Physiologically, *Idit*-deficient flies showed reduced exercise performance and defective cold resistance, which were rescued by exogenous expression of *Idit*. Exercise training increased endurance in wild-type flies, but not in *Idit*-deficient flies. Conversely, Idit is induced upon exercise training, and transgenic expression of *Idit* in wild-type flies increased endurance to the level of exercise trained flies. Finally, *Idit* deficiency prevented both exercise-induced increase in cardiac Atg8 and exercise-induced cardiac stress resistance, suggesting that cardiac autophagy may be an additional mechanism by which *Idit* is involved in the adaptive response to exercise. Our work suggests an ancient role of an Iditarod/Irisin/FNDC5 family of proteins in autophagy, exercise physiology, and cold adaptation, conserved throughout metazoan species.

## Significance

This study identifies *Iditarod* (*Idit*), a Drosophila gene that is similar to mammalian FNDC5/Irisin protein, as a regulator of autophagy, exercise performance, and cold resistance. Mammalian FNDC5/Irisin was previously shown to be implicated in exercise physiology. Our findings reveal that the role of Idit/Irisin/FNDC5 family proteins is conserved across animal species, including invertebrates. In flies, Idit deficiency led to impaired exercise endurance and cold tolerance, while Idit overexpression increased exercise endurance. Additionally, Idit was necessary for exercise-induced cardiac autophagy and stress resistance. This work suggests that the Idit/Irisin/FNDC5 family has ancient roles in autophagy, exercise physiology, and cold adaptation, providing insights into the conserved functions and mechanisms of these proteins across metazoan species.

Physical movement is essential for animal life; it provides means to acquire food sources, escape from danger, and find reproductive partners. Regular exercise is critical for maintaining physical endurance, not only in mammalian organisms but also in invertebrate organisms such as *Drosophila* (1, 2) and *Caenorhabditis elegans* (3, 4). Therefore, the benefits of exercise are widely conserved across different animal species.

Irisin is a small, exercise-inducible peptide that is processed from a precursor FNDC5 protein. Expression of FNDC5 is up-regulated upon exercise through PGC-1α (5), and the cleavage of the Irisin domain from FNDC5 is thought to be promoted by the physical movement of muscle (6). Although there was initial skepticism about the physiological importance of Irisin/FNDC5 in exercise physiology (7), extensive studies suggest that Irisin/FNDC5 is indeed important for producing multiple benefits of exercise, including browning of adipose tissue (5), bone strengthening (8, 9), and improvements in cognitive function (10, 11). Still, it is possible that FNDC5 functions in its intact unprocessed form (12), as FNDC5 also has a tissue-autonomous role in up-regulating autophagy and reducing fat accumulation in liver (13). The function of Irisin/FNDC5 has been predominantly studied in human cells or mouse models thus far. Studies in invertebrate model animals could provide valuable information about the evolutionarily conserved functions of the protein family and whether the central role of Irisin/FNDC5 in metabolic regulation associated with exercise is conserved across animal species. However, no Irisin/FNDC5 homolog had been found in the genome of invertebrate species.

During our genetic screening to isolate autophagy regulators, we serendipitously isolated a *Drosophila* Irisin/FNDC5 homolog, which we named *Iditarod* (*Idit*) because it is necessary for exercise endurance and cold resistance (Iditarod is a sled dog race covering ~1,000 miles with wind chill down to −100°F/−73 °C). Endogenous Idit was necessary for the Atg1/Atg13 complex to produce excessive autophagy and autophagic cell death, while *Idit* overexpression produced ectopic autophagy in diverse tissues including fat bodies, developing imaginal discs, and skeletal muscle. Most interestingly, *Idit*-deficient flies showed several phenotypes that are consistent with the role of Irisin/FNDC5 in mammalian systems, such as defective running endurance, impaired response to exercise training, and decreased cold resistance. These results suggest that the function of Irisin/FNDC5/Idit in regulating autophagy, exercise physiology and cold tolerance is conserved throughout the animal kingdom.

## Results

### Isolation of *Iditarod* (*Idit*) as a Suppressor of Atg1-Atg13-Induced Eye Degeneration.

**Fig. 1.**
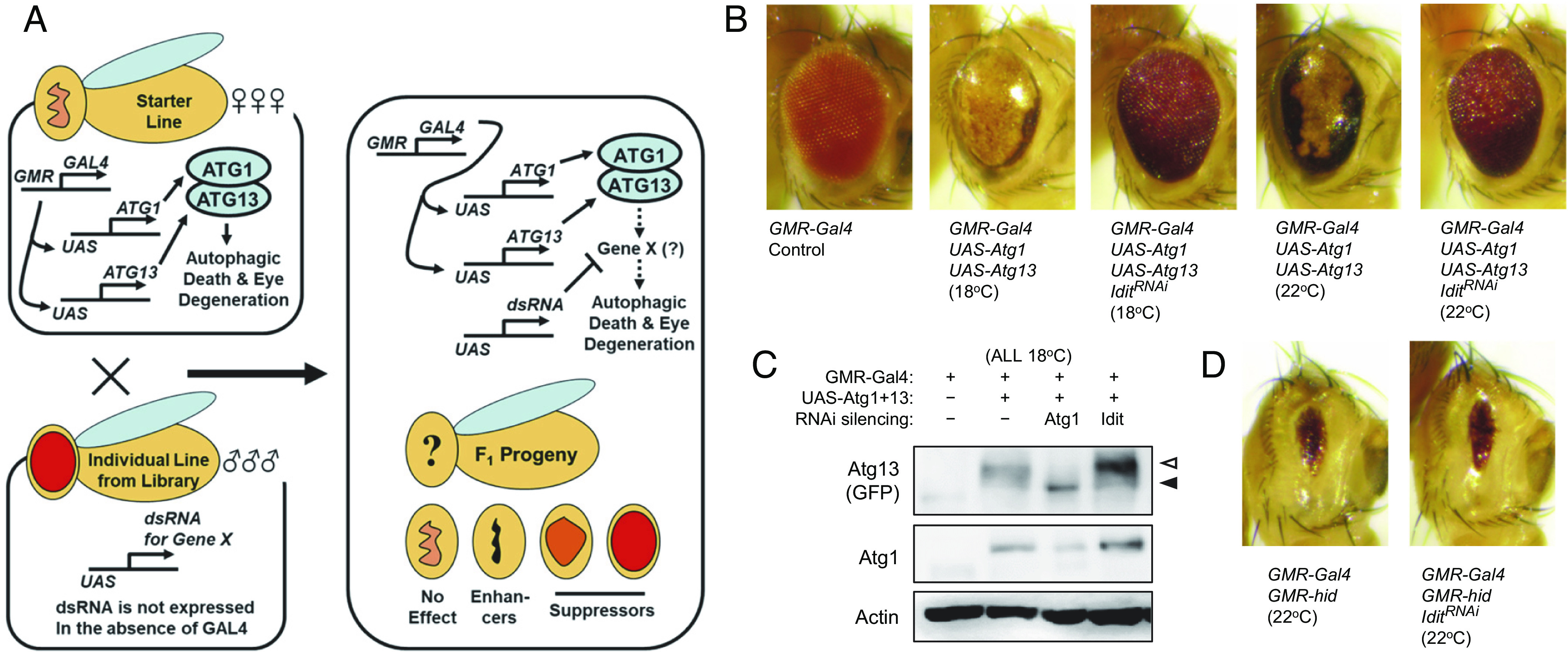
Isolation of *Idit* RNAi as a genetic modifier of the Atg1-Atg13-gain-of-function eye phenotype. (*A*) Schematic representation of the genetic screening for genetic modifiers of Atg1 and Atg13 in *Drosophila*. (*B*) Eyes from the flies expressing indicated transgenes were imaged using light dissection microscopy. (*C*) Eyes from the flies expressing indicated transgenes were subjected to immunoblotting examining indicated proteins. Open arrow indicates phosphorylation-induced electromobility retardation, which shifts the Atg13 band from its unphosphorylated location (closed arrow). (*D*) Eyes from the flies expressing indicated transgenes.

In the context of our previous work on autophagy regulation in *Drosophila* (14, 15), we performed a genetic screen using a transgenic RNAi library (16) to isolate regulators of autophagy (Fig. 1*A*). In this screening scheme, an autophagy-initiating protein kinase complex, composed of Atg1 and Atg13, was overexpressed in the *Drosophila* eye, resulting in a massive induction of autophagic cell death, leading to the complete degeneration of the ommatidia structure. Since eye degeneration does not abolish an organism’s viability or fertility, we were able to construct a stable *Drosophila* line that eye-specifically expresses Atg1-Atg13. The line was crossed to Transgenic RNAi Project (TRiP) stocks, with the hope that inhibition of genes essential for Atg1-Atg13-dependent autophagy would suppress the eye degeneration phenotype (Fig. 1*A*).

An RNAi line targeting an uncharacterized gene *CG33143* was isolated as a suppressor of the Atg1-Atg13 eye phenotype; we renamed *CG33143* as *Iditarod* (*Idit*). Although expression of Atg1 and Atg13 completely degenerated the eye structure, expression of *Idit^RNAi^* restored the normal eye size, and noticeably reestablished the ommatidial structure, when cultured at both 18 °C and 22 °C (Fig. 1*B*). Such strong suppression was not conferred by control RNAi transgenes (*SI Appendix*, Fig. S1*A*), such as the ones targeting *luciferase*, *mCherry*, and *LexA*.

The suppression of *GMR>Atg1+Atg13* phenotypes by *Idit^RNAi^* was not due to the inhibition of transgene expression, as evidenced by the western blot analysis of the eyes, which showed that Atg1 and Atg13 proteins were expressed at the same or increased levels in *Idit^RNAi^*-expressing flies compared to the control flies that do not express *Idit^RNAi^* (Fig. 1*C*). Notably, Atg1-induced Atg13 phosphorylation, monitored through electromobility changes in the SDS-PAGE gel (gel shift; indicated by the open arrowhead in Fig. 1*C*), was not suppressed by *Idit^RNAi^* expression, indicating that *Idit^RNAi^* inhibits the downstream effects of Atg1-Atg13-dependent autophagy without altering Atg1 expression or catalytic activities.

### Inhibition of *Idit* Suppresses Autophagic Cell Death but not Apoptosis.

Although Atg1-Atg13 overexpression induces massive autophagic cell death (17, 18), expression of *Idit^RNAi^* strongly suppressed excessive death and restored the cell death level close to the levels of either the control eye or the *Atg1^RNAi^*-expressing eye (*SI Appendix*, Fig. S2*A*). This suggests that *Idit^RNAi^* inhibits Atg1-Atg13-induced autophagic cell death. To determine whether *Idit^RNAi^* specifically suppresses autophagy-induced eye degeneration and cell death, we investigated the effect of *Idit^RNAi^* in apoptotic eye degeneration induced by *hid* (caspase activator). *Idit^RNAi^* expression did not alter *hid*-induced eye degeneration (Fig. 1*D*), indicating that *Idit^RNAi^* specifically inhibits autophagic cell death but not general apoptosis.

### Inhibition of *Idit* Suppresses Autophagy-Related Activities.

We monitored various autophagy-related activities in the eye tissue expressing Atg1-Atg13 and *Idit^RNAi^*. As expected, Atg1-Atg13 expression prominently up-regulated autolysosome formation, monitored through lysotracker, which is consistent with excessive autophagic activity (*SI Appendix*, Fig. S2*B*). Atg1-Atg13 also up-regulated Atg9 trafficking, as manifested by the increased number and intensities of Atg9 puncta (*SI Appendix*, Fig. S2*C*). *Idit^RNAi^* suppressed Atg1-Atg13-induced lysotracker staining (*SI Appendix*, Fig. S2*B*) and Atg9 puncta formation (*SI Appendix*, Fig. S2*C*), suggesting that *Idit^RNAi^* is critical for Atg1-Atg13 to produce excessive autophagy.

We also stained the eye tissue with endogenous Atg8, a marker for autophagosomes. The autophagic activity, monitored by Atg8 puncta, was very low in wild-type eye imaginal disc and only barely detectable around the morphogenetic furrow (gray bracket in *SI Appendix*, Fig. S2*D*, first column). However, when Atg1-Atg13 was expressed, prominent amounts of Atg8 accumulated in *GMR*-expressed postmorphogenetic furrow regions (white bracket in *SI Appendix*, Fig. S2*D*, second column). This was suppressed by *Idit^RNAi^* (*SI Appendix*, Fig. S2*D*, third column), but not a control *mCherry^RNAi^* (*SI Appendix*, Fig. S2*D*, fourth column). The results were quantified (*SI Appendix*, Fig. S2*E*), and the results clearly show that the induction of Atg8-positive puncta is substantially suppressed by *Idit* silencing, but not by *mCherry* silencing.

The *Atg13* transgene that we used is tagged with GFP, so we can monitor the behavior of Atg13-GFP. As expected, Atg13-GFP is strongly expressed by *GMR* driver in the postmorphogenetic furrow (*SI Appendix*, Fig. S2*D*, second column). There was a time lag between GMR expression and autophagy induction because we found a postmorphogenetic area where Atg13-GFP is expressed but Atg8 puncta is not prominent (black brackets in *SI Appendix*, Fig. S2*D*, second column). Interestingly, in the area of prominent Atg8 puncta induction, the amount of Atg13-GFP was markedly reduced (white brackets in *SI Appendix*, Fig. S2*D*, second column), consistent with the previous findings indicating that active autophagy turns over the Atg1-Atg13 protein complex (19–21). Atg8 puncta induction in this area, as well as Atg13-GFP reduction, were less pronounced in eye discs expressing *Idit^RNAi^* (white brackets in *SI Appendix*, Fig. S2*D*, third column). Consistent with these histological observations, the amount of Atg1 and Atg13 proteins in *GMR>Atg1+Atg13* fly eyes was noticeably up-regulated by *Idit^RNAi^* expression in western blot analysis as well (Fig. 1*C*). In contrast, *mCherry-RNAi* expression did not alter these regulations (white brackets in *SI Appendix*, Fig. S2*D*, fourth column). All these observations were quantified and determined to be statistically significant (*SI Appendix*, Fig. S2 *E* and *F*).

### *Idit* Encodes Drosophila Irisin/FNDC5 Homolog.

**Fig. 2.**
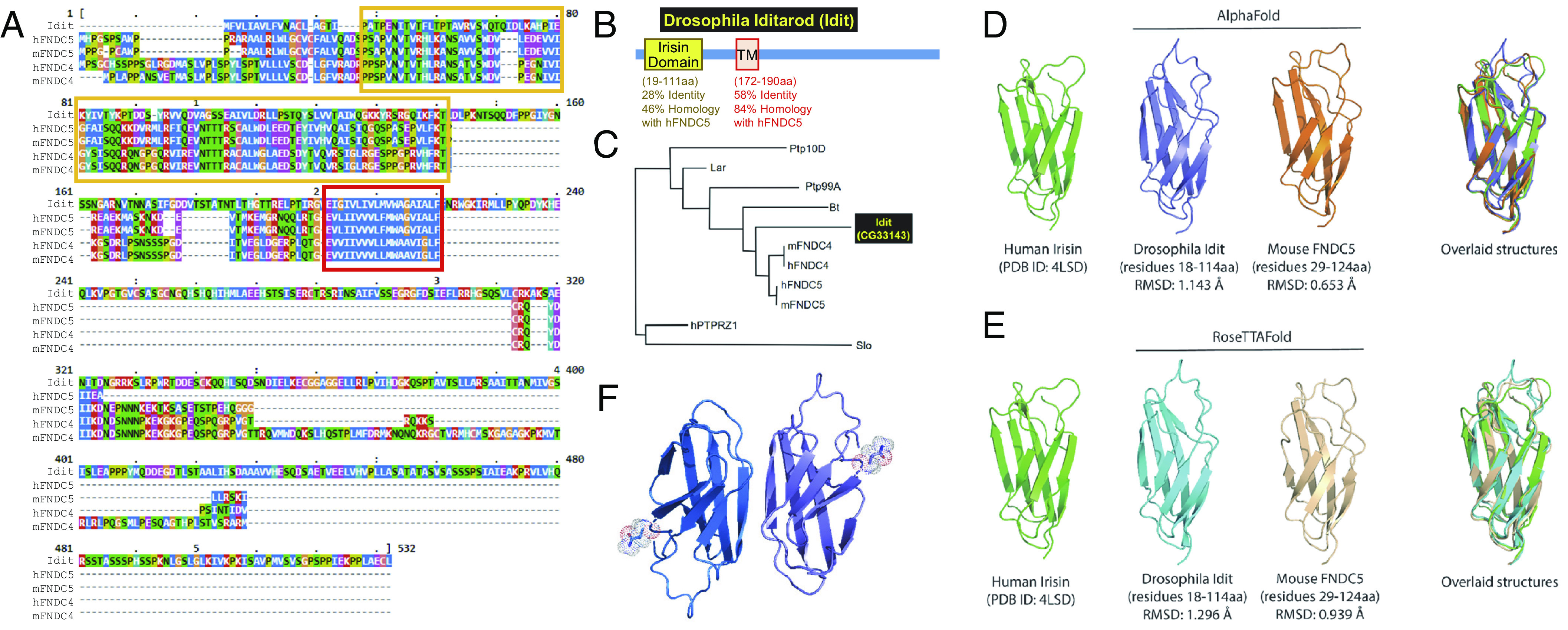
*Idit* encodes *Drosophila* homolog of Irisin/FNDC5. (*A*) Multiple sequence alignment using *Drosophila* Idit, human and mouse FNDC5, and human and mouse FNDC4, conducted with ClustalW. Hydrophobic residues are shaded in blue, polar residues in green, acidic residues in purple, basic residues in red, cysteine in orange, glycine in tan, proline in light green. Irisin domain is indicated by yellow box. Transmembrane domain (TM) is indicated by red box. (*B*) Idit has two domains that show substantial homology to human FNDC5—extracellular Irisin domain and TM domains. Homology of each domain to human FNDC5 is assessed after Needleman–Wunsch alignment of each domain. For additional protein domains, see *SI Appendix*, Fig. S3*C*. (*C*) Phylogenetic tree constructed using different proteins possessing Irisn/FNDC5-like sequences, conducted with PhyML. *Drosophila*, human and mouse species are identified as prefixes d, h, and m, respectively. (*D* and *E*) AlphaFold (*D*) and RoseTTAFold (*E*) predictions of Irisin homology domains from mouse FNDC5 and *Drosophila* Iditarod, compared with each other and human Irisin crystal structure (PDB: 4LSD). Cα RMSD is used for measuring the structural similarity in between. (*F*) The potential dimer model of AlphaFold-predicted Iditarod overlaid on the human Irisin dimer. The predicted glycosylation site of Asn residues (NxT motif) are displayed as stick/dot model.

BLAST search of Idit against the human and mouse proteome identified FNDC5 and FNDC4 as the proteins exhibiting the highest sequence homology (Fig. 2*A* and *SI Appendix*, Fig. S3*A*). However, BLAST search of FNDC5 against *Drosophila* proteome identified multiple proteins with similar homology, including Bent (Bt), Leukocyte-antigen-related-like (Lar), Protein tyrosine phosphatase 10D (Ptp10D) and 99A (Ptp99A), and Slowpoke (Slo); therefore, to establish the homologous relationship, we performed detailed domain and phylogenetic analysis. Domain analysis showed that Idit had the strongest homology with human FNDC5 in the extracellular Irisin domain and transmembrane domain (Fig. 2*B*). Although the homology was lower in the other regions, such as in the Irisin cleavage domain (CL) and intracellular domains (I1 and I2), Needleman-Wunsch global alignment analysis suggests that these elements might be also conserved as well (*SI Appendix*, Fig. S3 *B*-S3 *C*).

Although the Idit domain structure is globally similar to FNDC4/FNDC5, containing only one Irisin domain, transmembrane domain, and intracellular domain, other *Drosophila* proteins, such as Bt, Lar, Ptp10D, Ptp99A, and Slo, contained a number of other unrelated domains, such as immunoglobin, protein kinase, protein phosphatase, and ion channel domains (*SI Appendix*, Fig. S3*D*). Furthermore, when we performed phylogenetic analysis with FNDC5 and FNDC4 from mouse and human, along with all homologous *Drosophila* proteins, we identified that Idit is the most phylogenetically similar to FNDC5 and FNDC4 (Fig. 2*C*). These results were reproduced well when we used different tree construction algorithms, such as phylogenetic inferences using maximum likelihood (PhyML; Fig. 2*C*), as well as fast minimum evolution (*SI Appendix*, Fig. S3*E*) and cobalt dendrogram (*SI Appendix*, Fig. S3*F*) methods.

### Irisin Domain of Idit is Predicted to be Structurally Similar to Human/Mouse Irisin.

Recent improvements in AI-based structure prediction (22, 23) have dramatically improved the precision and efficiency of sequence-based structural determination. We used the two most advanced structure prediction methods, AlphaFold (Fig. 2*D*) (22) and RoseTTAFold (Fig. 2*E*) (23), to understand the structural relationship between Idit and human/mouse Irisin proteins. Both methods suggested the high structural homology of *Drosophila* Idit to the previously determined crystal structure of human Irisin [PDB ID: 4LSD, left panel (24)] as well as to the predicted model of mouse Irisin (Fig. 2 *D* and *E*). Since human Irisin forms a dimer, we expect that *Drosophila* Idit also functions as a dimer with a potential glycosylation site (NxT motif) that is also similar to human Irisin (Fig. 2*F*) (24).

The *Drosophila* genome contains another gene that has been putatively annotated as a FNDC5 homolog, *CG12541*. The CG12541 protein domain structure indeed showed a FNDC5-like domain (*SI Appendix*, Fig. S3*D*). However, in all phylogenetic analyses, CG12541 was isolated as an outgroup, and only distantly related to the Idit/FNDC5-family proteins (*SI Appendix*, Fig. S3 *E* and *F*). Furthermore, AlphaFold structure prediction of CG12541 did not show any domains that are structurally related to FNDC4/FNDC5/Idit (*SI Appendix*, Fig. S3*G*); importantly, CG12541 does not contain the beta-sheet structure that is essential for forming the Irisin structural fold. Therefore, it is unlikely that CG12541 is a structural and functional homolog of human/mouse Irisin.

In mammals, it was suggested that Irisin domain is cleaved from the membrane during exercise through a poorly defined mechanism that might involve physical shearing (25, 26). The exact site of cleavage is also ambiguous; however, it is thought to occur around the Irisin tail region (ending with DEVTMKE in human FNDC5). In multiple alignment analysis involving Drosophila Idit, FNDC5 proteins from human, mouse and zebrafish, and FNDC4 from human and mouse, it was shown that Idit sequence around the cleavage region (DDVTSTA in Idit) is more similar to FNDC5 than to FNDC4 (red box in *SI Appendix*, Fig. S3 *A*, *B*, and *H*).

Taken together, these conserved structural similarities suggest that Idit is the single homolog of Irisin/FNDC5 in the *Drosophila* genome. Therefore, we asked whether Idit had physiological functions similar to vertebrate Irisin/FNDC5.

### *Idit* Overexpression Induces Ectopic Autophagy.

Using the full-length *Idit* cDNA clone, available at Drosophila Genome Resources Center, we constructed a transgenic fly strain (*UAS-Idit*), which can conditionally overexpress Idit protein. Since Idit was necessary for Atg1-Atg13-induced autophagy (Fig. 1 and *SI Appendix*, Fig. S2), we were curious if Idit upregulation was sufficient for autophagy induction. *Idit* expression in fat body clones (marked by GFP expression) induced autophagy, as monitored by elevated Atg8 staining (*SI Appendix*, Fig. S4*A*). Also, when *Idit* was expressed in the dorsal wing compartment, wings were bent upward, indicating that dorsal wing growth is inhibited (*SI Appendix*, Fig. S4*B*). Lysotracker staining of the developing wing disc showed widespread induction of autophagy in the dorsal wing compartment, but not in the ventral wing compartment (*SI Appendix*, Fig. S4*C*). These results indicate that *Idit* overexpression induces ectopic autophagy in a cell- and tissue-autonomous manner.

It was recently shown that overexpression of FNDC5 in cultured human cells can up-regulate autophagic flux (27). Consistent with this, we found that overexpression of mouse FNDC5, as well as Idit, could up-regulate autophagic flux in human embryonic kidney 293 (HEK293) cells (*SI Appendix*, Fig. S4 *D*–*F*). Interestingly, even though C-terminal tagged proteins were able to up-regulate autophagy (*SI Appendix*, Fig. S4 *D*–*F*), N-terminal tagging, which can interfere with proper membrane topology by disrupting signal sequence, prevented expression of Idit (*SI Appendix*, Fig. S4*G*) and abolished mouse FNDC5’s abilities in up-regulating autophagic flux (*SI Appendix*, Fig. S4*H*). These suggest that the autophagy-up-regulating role is conserved between mammalian FNDC5 and Idit.

### Generation of *Idit* Knock-in Mutant and Its Revertant.

**Fig. 3.**
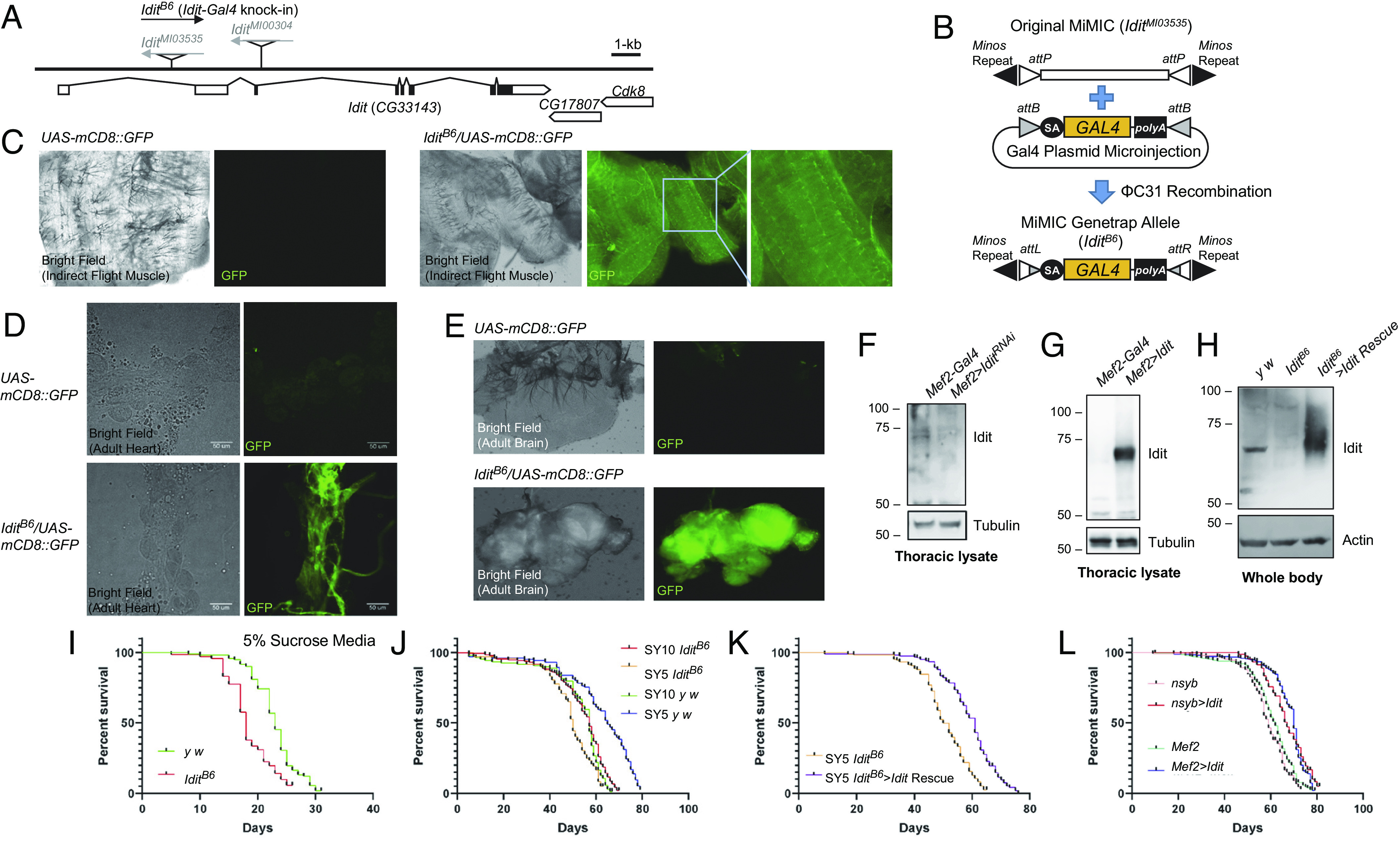
Generation and characterization of *Idit* gene-trap mutants. (*A*) Schematic genomic organization of the *Idit* (*CG33143*) locus and *Idit* mutants. Triangles indicate transposon insertions. Open boxes: untranslated exons of *Idit* or other genes; closed boxes: protein-coding exons of *Idit*; size bar: relative length of 1 kb genomic span. (*B*) Schematics of how we converted original MiMIC allele (*Idit^MI03535^*) into MiMIC gene-trap allele (*Idit^B6^*). (*C*–*E*) Strong *Idit^B6^>GFP* expression detected in adult skeletal muscle (*C*), heart (*D*) and brain (*E*) tissues. (*F*–*H*) Immunoblot analysis of indicated proteins in fly tissues of indicated genotypes. (*I*–*L*) Lifespan analysis of flies with indicated genotype and treatment (*n* ≥ 70 for all groups presented). *P* < 0.001 between *y w* and *Idit^B6^* (*I* and SY5 in *J*), *Idit^B6^* and *Idit^B6^* Rescue (*K*), and control and *Idit*-overexpressing groups (*L*), in log-rank test.

*Idit* mutants were not previously reported or publicly available; however, two MiMIC (28) insertions, *Idit^MI03535^* and *Idit^MI00304^*, were available at Bloomington stock center (Fig. 3*A*). Both alleles did not show any noticeable phenotypes, and they expressed normal levels of *Idit*. MiMIC alleles, however, have the potential to be converted into an active genetrap allele so that other genetic elements, such as GAL4, are expressed instead of the endogenous gene in the targeted locus. Using microinjection, we replaced the MiMIC element with genetrap GAL4 constructs (Fig. 3*B*), leading to the generation of *Idit* targeted knock-in allele, *Idit^B6^* (Fig. 3*A*; see *Methods* for details in allele construction and screening). In this allele, GAL4, instead of Idit, is expressed under the control of the *Idit* promoter; therefore, when crossed to the *UAS-mCD8:GFP* transgenic line (mCD8 targets GFP to cell membrane), we can monitor the expression pattern of *Idit* through fluorescence microscopy. *Idit^B6^>UAS-mCD8:GFP* was expressed very highly in the adult skeletal muscle, heart, and brain (Fig. 3 *C–E*). Using cardiac and skeletal muscle-specific *Mef2-Gal4*, *Drosophila* thoracic muscle tissue, and rabbit antisera raised against Idit protein, we showed that *UAS-Idit^RNAi^* and *UAS-Idit* were effective in modulating the level of Idit protein in thoracic muscle (Fig. 3 *F* and *G*). In addition, we also showed that the *Idit^B6^* knock-in allele indeed abolished Idit expression and that breeding of *Idit^B6^* with *UAS-Idit* can restore Idit expression under the control of *Idit-*GAL4 encoded in *Idit^B6^*, providing transgenic revertants or rescue flies (Fig. 3*H*). Because of the signal amplification through the GAL4-UAS loop, the expression of Idit was higher in the *Idit^B6^>Idit* Rescue flies than in wild-type flies (Fig. 3*H*).

### *Idit*-Deficient Flies do not Gain Lifespan-Expanding Benefits from Dietary Restriction.

Circulating Irisin and muscle FNDC5 expression are influenced by dietary status and nutrient stress (29). Therefore, using the genetic tools we generated for Idit modulation, we investigated the role of Idit in the nutrient response and lifespan regulation processes. Like many other mutant strains with autophagy defects (30), *Idit^B6^* mutants were more sensitive to nutrient starvation (sucrose-only media) compared to control flies (Fig. 3*I*). In normal rearing conditions [10% sucrose–yeast (SY) media], *Idit^B6^* and its genetic background control (*y w*) showed an almost identical lifespan (Fig. 3*J*). However, interestingly, although control flies extended their lifespan in calorie-restricted conditions (5% SY media), *Idit^B6^* flies shortened their lifespan (Fig. 3*J*), indicating that Idit is important for getting the benefits of caloric restriction in flies. Such defects were suppressed by *Idit* transgenic rescue (Fig. 3*K*), indicating that *Idit* deficiency is indeed the cause of defective response to caloric restriction. In contrast, *Idit* overexpression in neurons (through *nsyb-GAL4*) or muscle (through *Mef2-GAL4*) slightly extended lifespan (Fig. 3*L*). These results collectively suggest that Idit is important for normal lifespan responses to nutritional modulations.

### *Idit* Deficiency Diminishes Physical Endurance.

**Fig. 4.**
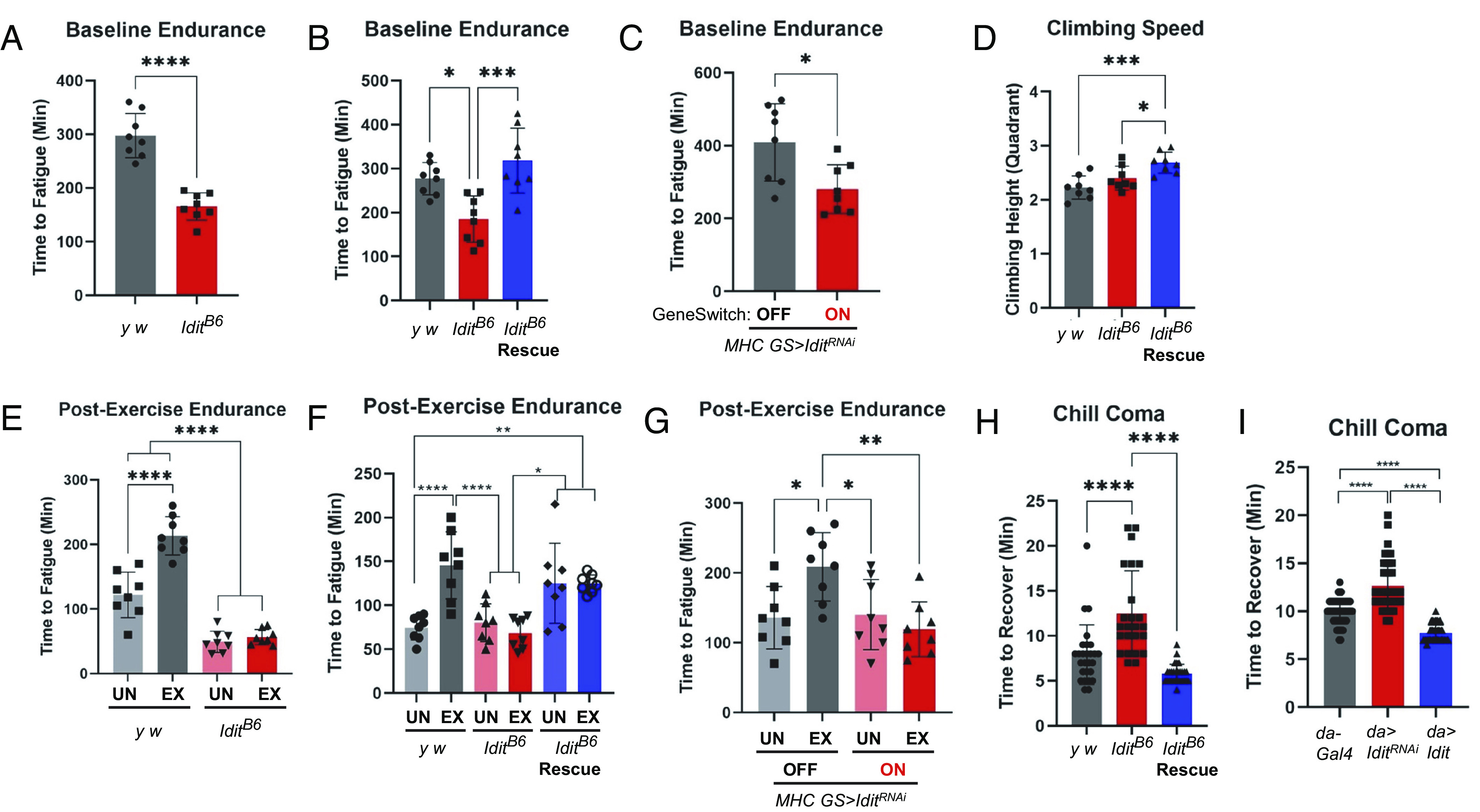
*Idit^B6^* mutants show impaired endurance and exercise response. (*A*–*C*) Baseline endurance at 5 d of age (*n* = 8; unpaired *t*-test for *A* and *C*, one-way ANOVA with Tukey test for post hoc pairwise comparisons for *B*). (*D*) Climbing speed measured as average quadrant height climbed in a 3-s negative geotaxis test (*n* = 8; one-way ANOVA). (*E–G*) Endurance of exercised (EX) or control (UN) flies at 25 d of age (*n* = 8; two-way ANOVA with Tukey multiple comparison; genotype by exercise interaction *P* < 0.01 for *E*–*G*). (*H* and *I*) Time to recovery following a 2-h chill coma (*n* = 24; one-way ANOVA). For *C* and *G*, *MHC-GS-Gal4* was used to inducibly drive *Idit* RNAi expression. “ON” indicates groups fed 100 µm mifepristone and “OFF” indicates groups fed vehicle solution. For *A*–*G*, *n* values for endurance represent the number of vials. Each vial contained 20 flies and was scored as fatigued when less than 4 flies responded to 3 consecutive stimuli. For *H* and *I*, *n* values indicate individual flies. Error bars represent SD. Asterisks indicate significance from one-way ANOVA, two-way ANOVA or unpaired *t* test as indicated above; *P < 0.05, **P < 0.01, ***P < 0.001, ****P < 0.0001.

Since mammalian Irisin/FNDC5 is induced by exercise and mediates some of its effects (5, 10, 11), we investigated the role of Idit in exercise performance by using a *Drosophila* model of endurance exercise (2). Compared to its wild-type counterpart, homozygous *Idit^B6^* mutants had dramatically reduced baseline climbing endurance (Fig. 4*A*), while heterozygotes were normal (*SI Appendix*, Fig. S1*C*). Endurance of *Idit^B6^* mutants was restored by transgenic expression of *Idit* (Fig. 4*B*). Muscle-specific silencing of Idit through *Idit^RNAi^* also impaired endurance to a level similar to that of *Idit^B6^* mutation (Fig. 4*C*). Although endurance is severely impaired by *Idit* deficiency, climbing speed was not diminished, indicating that *Idit* mutation does not directly impair flies’ mobility (Fig. 4*D*), but specifically diminishes endurance.

### *Idit* Deficiency Nullifies Exercise Effect on Endurance Extension.

We have formerly shown that exercise training can extend endurance in *Drosophila* (31–35). We examined the impact of *Idit* mutation on the endurance-extending effects of chronic exercise. Although exercise substantially improved endurance in control flies, neither *Idit^B6^* mutants nor flies expressing *Idit^RNAi^* in muscle adapted to chronic exercise. Instead, both retained low endurance (Fig. 4 *E* and *G*). Interestingly, transgenic expression of *Idit* increased endurance of 25-d-old *Idit^B6^* mutants, regardless of exercise treatment. Indeed, *Idit^B6^* transgenic rescue flies showed endurance comparable to exercised wild-types of the same age (Fig. 4*F*). These results indicate that Idit is essential for producing endurance-extending effects in response to chronic exercise.

### *Idit* Deficiency Reduces Cold Resistance.

Interestingly, reminiscent of the role of mammalian Irisin/FNDC5 in controlling thermogenesis and cold tolerance (5, 36), *Idit^B6^* mutant flies displayed impaired cold resistance, while transgenic rescue of *Idit* expression restored cold resistance (Fig. 4*H*). Also, with *da-Gal4* driver inducing ubiquitous transgenic expression, *Idit^RNAi^* impaired cold resistance while *Idit* upregulation strengthened it (Fig. 4*I*).

### *Idit* Expression is Induced by Chronic Exercise or PGC-1α Activation to Increase Endurance.

**Fig. 5.**
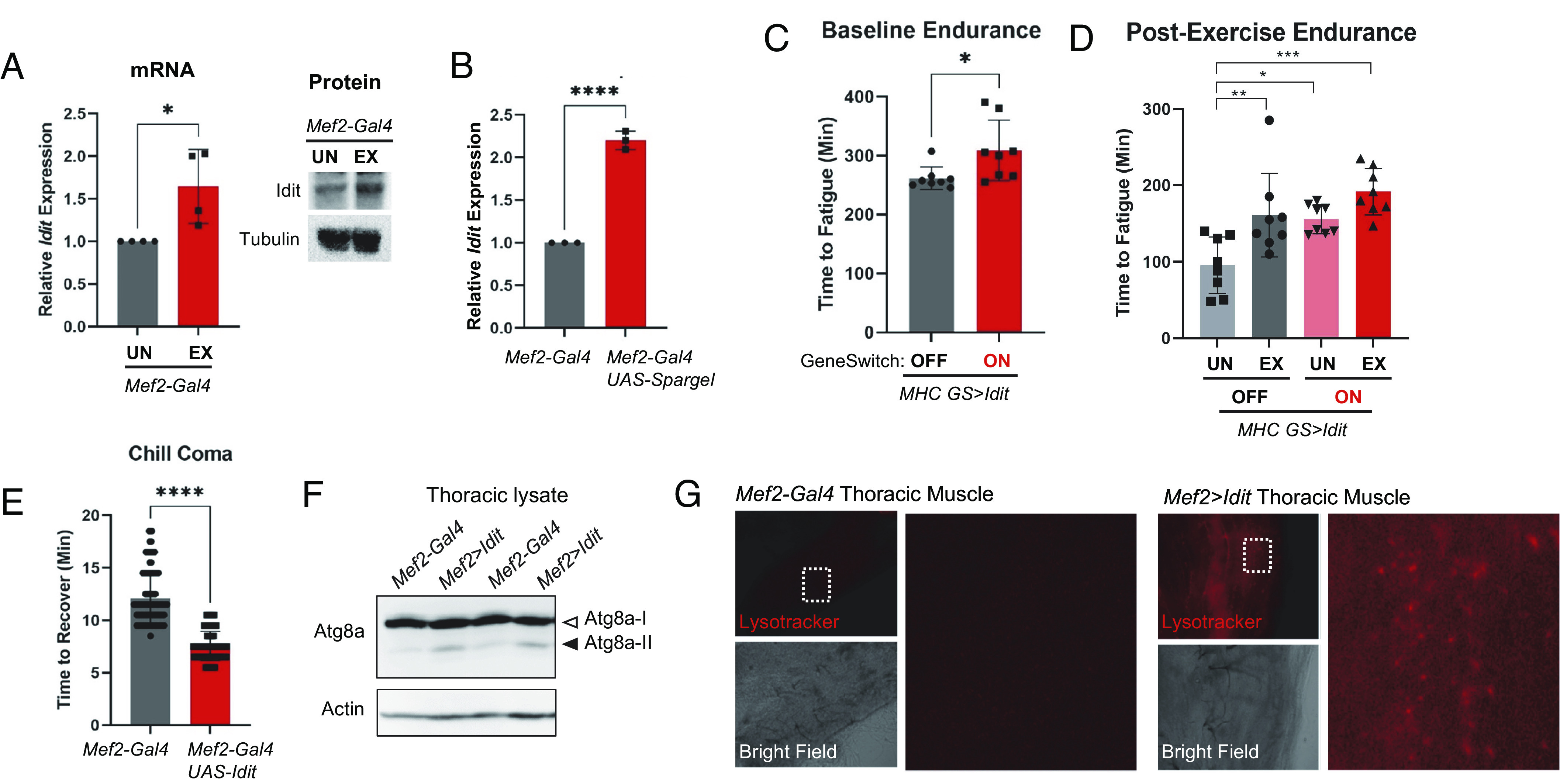
Induction of *Idit* expression is sufficient to improve endurance. (*A* and *B*) *Idit* mRNA expression was measured by quantitative RT-PCR. Measurements took place in outcrossed *Mef2-Gal4* flies to better match the genetic background of overexpression experiments in *B* (*n* = 3; each set includes 10 flies). For *A*, exercised (EX) or control-treated (UN) flies were examined at 25 d of age. For *B*, unexercised flies were examined at 5 d of age. (*C*) Baseline endurance was measured at 5 d of age (*n* = 8; unpaired *t*-test). *MHC-GS-Gal4* was used to control *Idit* expression. ON indicates groups fed 100 µm mifepristone and OFF indicates groups fed vehicle solution. (*D*) Endurance of exercised (EX) or control (UN) flies was measured at 25 d of age (*n* = 8; two-way ANOVA with Tukey multiple comparison; genotype by interaction effect *P* = 0.27, genotype effect *P* = 0.002, exercise effect *P* = 0.0007). (*E*) Time to recovery following a 2-h chill coma (*n* = 24; unpaired *t*-test). (*F*) Immunoblot analysis of indicated proteins in fly tissues of indicated genotypes. Open and closed arrows indicate unprocessed (Atg8a-I) and processed (Atg8a-II) Atg8a proteins, respectively. (*G*) Lysotracker analysis of indirect flight muscle from flies of indicated genotypes. Brightfield images corresponding fluorescence image was shown below. Boxed areas are magnified in *Right*. Error bars represent SD. Asterisks indicate significance from one-way ANOVA, two-way ANOVA, or *t* test as indicated above; *P < 0.05, **P < 0.01, ***P < 0.001, ****P < 0.0001.

Mammalian Irisin/FNDC5 is induced in skeletal muscle upon exercise or PGC-1α activation (5). Likewise in flies, we found that *Idit* expression is up-regulated by both exercise (Fig. 5*A*) and muscle-specific *dPGC1/Spargel* induction (Fig. 5*B*). Because *Idit* is induced upon exercise and because transgenic *Idit* expression extended endurance of *Idit^B6^* mutants up to the exercised wild-type level even without exercise training, we asked whether *Idit* induction is sufficient to extend endurance of otherwise wild-type flies. Muscle-specific transgenic overexpression of *Idit* indeed produced small but statistically significant improvements in endurance (Fig. 5*C*), with no further benefit from exercise training (Fig. 5*D*). To assess the degree of phenotypic conservation with mammalian FNDC5, we expressed the mouse FNDC5 (see *Methods* for construction of fly line) in fly muscle. We found that muscle-specific overexpression of *mFNDC5* did not extend endurance in a wild-type background (*SI Appendix*, Fig. S1*B*), but expression of *mFNDC5* in muscle was able to rescue endurance of *Idit* homozygous mutants to the level that is indistinguishable from the heterozygotic control flies (*SI Appendix*, Fig. S1*C*), indicating significant phenotypic conservation across species. Muscle-specific *Idit* overexpression was also sufficient to improve cold resistance of the whole organism (Fig. 5*E*). Muscle-specific *Idit* overexpression induced ectopic autophagy in skeletal muscle, as assessed by Atg8 processing (Fig. 5*F*) and lysotracker staining (Fig. 5*G*).

### *Idit* Mutants do not Increase Cardiac Atg8 Following Chronic Exercise Training.

**Fig. 6.**
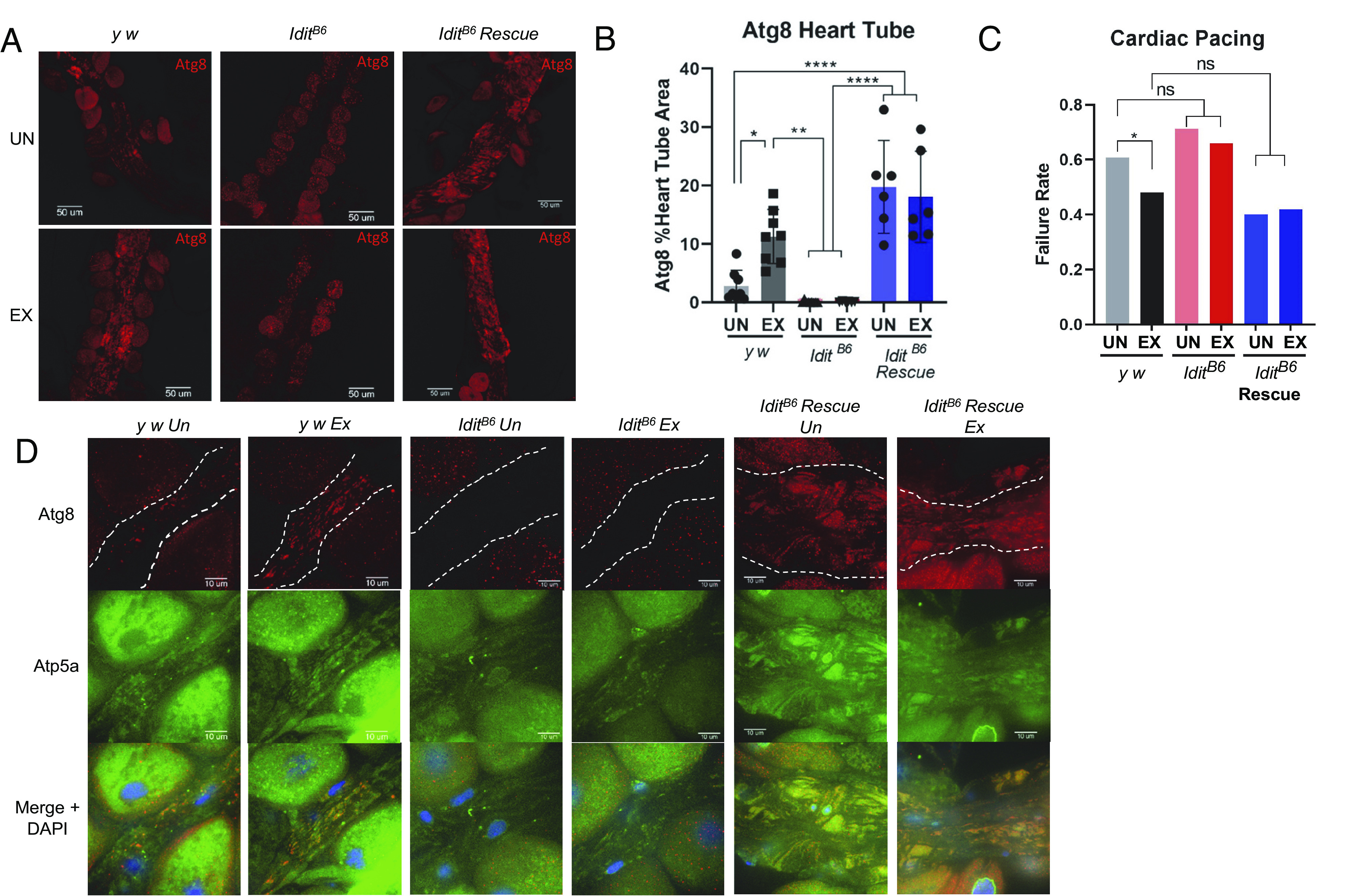
(*A*) Atg8 staining in dissected hearts from exercised (EX) and unexercised (UN) flies after 3-wk exercise protocol at 25 d old. Images are 20× magnification. Scale bar represents 50 µm. (*B*) Cardiac Atg8 fluorescence quantification from *A* (*n* ≥ 6, two-way ANOVA with Tukey multiple comparison, genotype by exercise interaction effect *P* = 0.01). (*C*) Hearts of flies were paced at 6 Hz for 30 s and immediately assessed visually for arrest or fibrillation, with either event noted as failure and the failure rate being the percentage of hearts that were in arrest or fibrillation after stimulus (*n* > 79, χ^2^ = 29.20, df = 5; *P* < 0.0001, Chi-square test, *y^1^w^1^* ex vs. *y^1^w^1^* un *P* = 0.04). (*D*) Dissected hearts of exercised and unexercised flies after 3 wk of exercise protocol stained for Atg8 (red) to mark autophagy and Atp5a (green) to mark mitochondria. Nuclei stained with DAPI. Heart tube is outlined with dotted white lines. Images are 100× magnification and scale bar represents 10 µm. Error bars represent SD. Asterisks indicate significance from two-way ANOVA in *B*; chi-square analysis for binary variables in *C*; ns = not significant. *P < 0.05, **P < 0.01, ****P < 0.0001.

Since endurance exercise promotes cardiac autophagy in mammals (37), we next asked if cardiac autophagy was altered during chronic exercise in *Idit^B6^* mutants. Following 3 wk of exercise training, Atg8 staining was significantly higher in the heart tubes of exercised wild-type flies than in unexercised. Atg8 staining in hearts of *Idit^B6^* mutants was absent, regardless of exercise status, indicating a reduction in autophagy machinery. Conversely, cardiac Atg8 staining was restored in both exercised and unexercised groups of *Idit^B6^* transgenic rescue flies (Fig. 6 *A* and *B*), which is consistent with the endurance rescue phenotype (Fig. 4*F*).

Since *Idit^B6^* mutants had a strong reduction in cardiac Atg8, we asked if this affected cardiac function by pacing the hearts of the flies using a previously established cardiac stress protocol (38). We found that the hearts of wild-type exercised flies failed less under pacing stress than unexercised, consistent with previous reports (1, 32). Both exercised and unexercised *Idit^B6^* mutants failed at the same rate as unexercised wild-type flies and *Idit^B6^* transgenic rescue flies failed at the same rate as wild-type exercise flies (Fig. 6*C*). These results are consistent with the endurance data (Fig. 4*F*) and indicate an association between cardiac Atg8 expression, cardiac function, and postexercise endurance.

Mitochondria-specific autophagy, or mitophagy, in cardiomyocytes is recognized as an adaptive response to chronic exercise training (37), as it is important to maintain a pool of healthy, functional mitochondria that can efficiently produce energy to meet the energy demands of exercise. To assess a possible role of *Idit* in mitophagy, we costained dissected heart tubes with Atg8 and Atp5a to mark autophagy and mitochondria, respectively, and examined if these two markers colocalized with fluorescence microscopy. We found that Atg8 localized to mitochondria more in exercised wild-type flies compared to unexercised. *Idit^B6^* hearts had virtually no colocalization due to diminished Atg8 expression. Conversely, *Idit^B6^* transgenic rescue hearts displayed more colocalization of Atg8 to mitochondria regardless of exercise status (Fig. 6*D*). While these data do not definitively measure mitophagy, they are consistent with the idea that *Idit* may stimulate mitophagy during chronic exercise.

## Discussion

Here, we isolated *Idit* in a screen for regulators of autophagy initiated by the Atg1 complex. Atg1 (known as ULK1/2 in mammals) is the first gene that was isolated as an autophagy regulator (39). Atg1 is a protein kinase and forms a complex with Atg13 and Atg17 (known as Fip200 in mammals) that is essential for autophagy initiation (40). Based on the epistatic relationship with other autophagy-regulating components, the Atg1 complex is thought to be one of the most upstream initiators of autophagy signaling. Indeed, multiple studies have shown that Atg1 is essential for initiating autophagy in response to stress inputs such as nutritional starvation and oxidative stress induction (41, 42). These stress inputs produce Atg1 activation through multiple mechanisms, such as decreased mTORC1-induced inhibitory phosphorylation and increased AMPK-induced activatory phosphorylation (43). Once activated, Atg1 phosphorylates multiple proteins controlling autophagy; in mammals, Atg1 phosphorylates Beclin (41, 44) and Atg14 (45), which together activates the class III PI3K complex for autophagy initiation. Still, the mechanistic details of how Atg1 instigates autophagy are not completely understood.

Due to its strong autophagy-initiating capabilities, concomitant overexpression of Atg1 and Atg13 strongly activates autophagy, leading to excessive autophagy and autophagic cell death (18). Silencing of Idit attenuated such excessive autophagy phenotypes, suppressing autolysosomal expansion, Atg8 puncta accumulation, and inhibiting autophagic cell death. Expression of Atg1 and Atg13 was not reduced, and Atg1-dependent Atg13 phosphorylation was not inhibited, indicating that *Idit* silencing does not reduce Atg1 activity and rather inhibits downstream signaling of Atg1. *Idit* silencing also reduced Atg9 puncta formation and Atg13 turnover. These results suggest that Idit is necessary for Atg1-Atg13-induced autophagy induction. We also performed gain-of-function experiments by overexpressing *Idit* in different tissues. In fat bodies, developing wing imaginal discs, and adult skeletal muscle, Idit cell- and tissue-autonomously induced autophagy. Therefore, Idit seems to be an essential component of Atg1-dependent autophagy that is also sufficient to induce autophagy when highly expressed.

Considering that Idit, like its mammalian counterpart, Irisin/FNDC5, is expected to be a transmembrane protein, its involvement in cell-autonomous autophagy signaling was quite unexpected. Still, studies from mouse liver suggest that Irisin/FNDC5 activates AMPK and inhibits mTORC1 to up-regulate autophagy (13). It is possible that Irisin/FNDC5 produces effects through autocrine signaling, since cultured muscle cells treated with recombinant Irisin increase AMPK and ACC phosphorylation (46). However, considering that both AMPK and mTORC1 control autophagy through Atg1 (43), our data indicating that Idit controls autophagy downstream of Atg1 suggest that there are additional mechanisms conferred by Idit that contribute to the autophagy control. For instance, it is possible that membrane-bound Idit is important for membrane trafficking that is necessary for autophagosome formation. It is also possible that the cytoplasmic domain of Idit, which contains sequence motifs distantly related to human FNDC5, somehow instigates signaling to up-regulate autophagic activities in cells. The mechanism of how Idit induces autophagy requires further investigation.

It is well established that exercise induces autophagy in cardiac and skeletal muscle (47) and is essential for proper muscle function (48). Considering the involvement of Irisin/FNDC5 in mammalian exercise physiology, we were curious about the comparable role of Idit in *Drosophila*. Notably, exercise in *Drosophila* produces physiological effects similar to mammals, such as increased endurance and speed and improved mitochondrial health and cardiac performance (33, 49). The role of genetic components controlling exercise physiology, such as PGC-1α (50), Sestrin (34, 51), and adrenergic signaling pathways (31, 32), is also highly conserved between mammals and *Drosophila*. In mammals, Irisin/FNDC5 was shown to be necessary for exercise-induced browning of adipose tissue (5), upregulation of glucose uptake in skeletal muscle (46), maximum oxygen consumption (52), and improvements of bone strength (8, 9) and brain function (10, 11). Similar to these characterized functions of Irisin/FNDC5, we show that Idit in *Drosophila* is necessary for normal physical endurance, as well as endurance-extending effects of exercise training. Importantly, both exercise and PGC-1α, the two signals that lead to Irisin/FNDC5 upregulation in mice (5), were able to up-regulate *Idit* in *Drosophila* muscle, and induction of *Idit* was sufficient to confer the endurance-extending effects of exercise training, even in unexercised flies. Exercise may also induce cleavage of Irisin domain from Idit, like human FNDC5, which may be important for systemic adaptation to exercise in different tissues. Further research is necessary to explore the possibility that cleaved Idit may perform functions similar to human Irisin.

Since Atg8 was up-regulated with Idit overexpression, we hypothesized that *Idit^B6^* mutants would have impaired cardiac autophagy in response to exercise. Indeed, we found that *Idit^B6^* mutants had a strong reduction of Atg8 in the heart tube similar to unexercised wild-type flies, while *Idit^B6^* transgenic rescue flies had Atg8 levels comparable to exercised wild-type. This is consistent with previous findings that show Irisin administration increased Atg8 in cultured rat cardiomyocytes (53). Furthermore, *Idit* expression was positively associated with postexercise endurance performance and cardiac stress resistance. This indicates that *Idit* is an important mediator of chronic exercise adaptations, and one mechanism for this may be by promoting autophagy, including mitophagy, in the heart. Irisin administration has been shown to mediate mitophagy in cultured cardiomyocytes in response to hypoxic stress through Opa1 (54). Consistent with this, we find in flies that Atg8 colocalization with Atp5a in cardiomyocytes is dependent on *Idit*.

Idit was also important for resistance against various stresses, such as nutritional deprivation and cold exposure, and necessary for nutrition-dependent regulation of lifespan. Of note, both Idit and Sestrins, two exercise regulators in *Drosophila*, are both found to be required for cold resistance [(55) and this study]. Both proteins are also involved in mammalian thermogenesis (5, 56, 57). This also suggests possible ancestral roles of these proteins in cold and exercise, which have been adapted in mammals to include fat thermogenesis and beiging of WAT.

In summary, here we isolated *Idit*, the *Drosophila* homolog of Irisin/FNDC5, and demonstrated its evolutionarily conserved functions in regulating autophagy and exercise physiology. These results suggest that Irisin/FNDC5 has an ancient role in controlling physical movements in the metazoan system and demonstrate that, in addition to the well-studied role of mammalian Irisin as a systemic hormone, Idit exhibits cell- and tissue-autonomous functions that are critical for regulation of autophagic metabolism. Further studies are warranted to examine the mechanistic connection between Idit and autophagy and how these pathways are coordinated during exercise training for stress resistance and endurance extension.

## Methods

### Drosophila Exercise model.

Flies were collected under light CO_2_ anesthesia within 2 h of adult eclosion and separated into vials containing 20 flies. Flies were then separated into two groups: exercised and unexercised groups. Both unexercised and exercised groups of flies were placed on the exercise training device at the same time to control for exercise-independent environmental factors. Every 15 s, the exercise device drops the vials of flies to induce an innate negative geotaxis response in a repetitive manner. Although exercised flies can run to the top of the vial, unexercised flies were prevented from running by a foam stopper placed low in the vial. Daily exercise time was gradually increased to generate a ramped program (1.5–2.5 h, 5 d/wk) that can improve mobility in flies. For all experiments in this study, males were used for all analyses as they are more responsive to exercise training. The exercise training was performed at the same time of day each day shortly after lights-on. Exercise protocol, including postexercise analyses such as runspan, cardiac pacing, and climbing speed assays, are described in further detail elsewhere (2).

### Sequence Alignment and Phylogeny.

Multiple sequence alignments and tree construction was performed using web-based Clustal Omega (58) and Mview (59) tools available at EMBL-EBI, Constraint-based Multiple Alignment (60) and Fast Minimum Evolution (61) tools available at NCBI. Global alignment between two sequences were performed using Needleman–Wunsch Global Alignment tool (62) available at NCBI. Specific methods to construct alignments and trees, and specific sequence segments used to calculate identity and similarity are indicated in the corresponding figure and figure legends.

### Structural Prediction and Analyses using AlphaFold and RoseTTAFold.

Amino acid sequences corresponding to the Irisn homology domains from Mouse FNDC5 (a.a. 29-124) and *Drosophila* Iditarod (a.a. 18-114) were subjected to AlphaFold and RoseTTAFold analyses using default parameters (22, 23). Structures were visualized and compared in PyMOL (Molecular Graphics System Version 2.0 Schrödinger LLC). Potential dimerization interaction and N-glycosylation sites (NxT motif) were visualized on the AlphaFold model. The complete models are available in ModelArchive at https://www.modelarchive.org/doi/10.5452/ma-u8lwn (*Drosophila* Iditarod-AlphaFold), https://www.modelarchive.org/doi/10.5452/ma-aq7lo (*Drosophila* Iditarod-RoseTTAFold), https://www.modelarchive.org/doi/10.5452/ma-6oxzk (Mouse FNDC5-AlphaFold), and https://www.modelarchive.org/doi/10.5452/ma-n3akt (Mouse FNDC5-RoseTTAFold). CG12541 model was retrieved from the Alphafold Protein Structure Database (https://alphafold.ebi.ac.uk/entry/Q9W3T1).

### Statistics.

For endurance, chill coma recovery, mRNA, and protein expression quantifications, a two-tailed Student *t* test was used to calculate the statistical significance of the differences between two groups, one-way ANOVA was used to test for statistical significance between three or more groups, and two-way ANOVA was used to compare multiple groups with multiple treatments (e.g., exercise and genotype effects). Two-way ANOVA was used to examine the cardiac Atg8 fluorescence quantification. One-way ANOVA was used to examine climbing speed. Log-rank analysis was used to examine survival and longevity assays. Chi-square test for binary variables was used to test for statistical significance in the cardiac pacing assay.

Description of fly and cell culture lines and care, qRT-PCR and westerns, and fly physiological assays can be found in *SI Appendix*.

## Supplementary Material

Appendix 01 (PDF)

## Data Availability

All study data are included in the article and/or *SI Appendix*.
