# Supplementary material for: Iditarod, a Drosophila homolog of the Irisin precursor FNDC5, is critical for exercise performance and cardiac autophagy
Source: Proc Natl Acad Sci U S A. 2023 Sep 18;120(39):e2220556120. doi: 10.1073/pnas.2220556120 (PMC10523451; doi:10.1073/pnas.2220556120)
Supplement: Supplementary file 1 — Appendix 01 (PDF) [file pnas.2220556120.sapp.pdf]

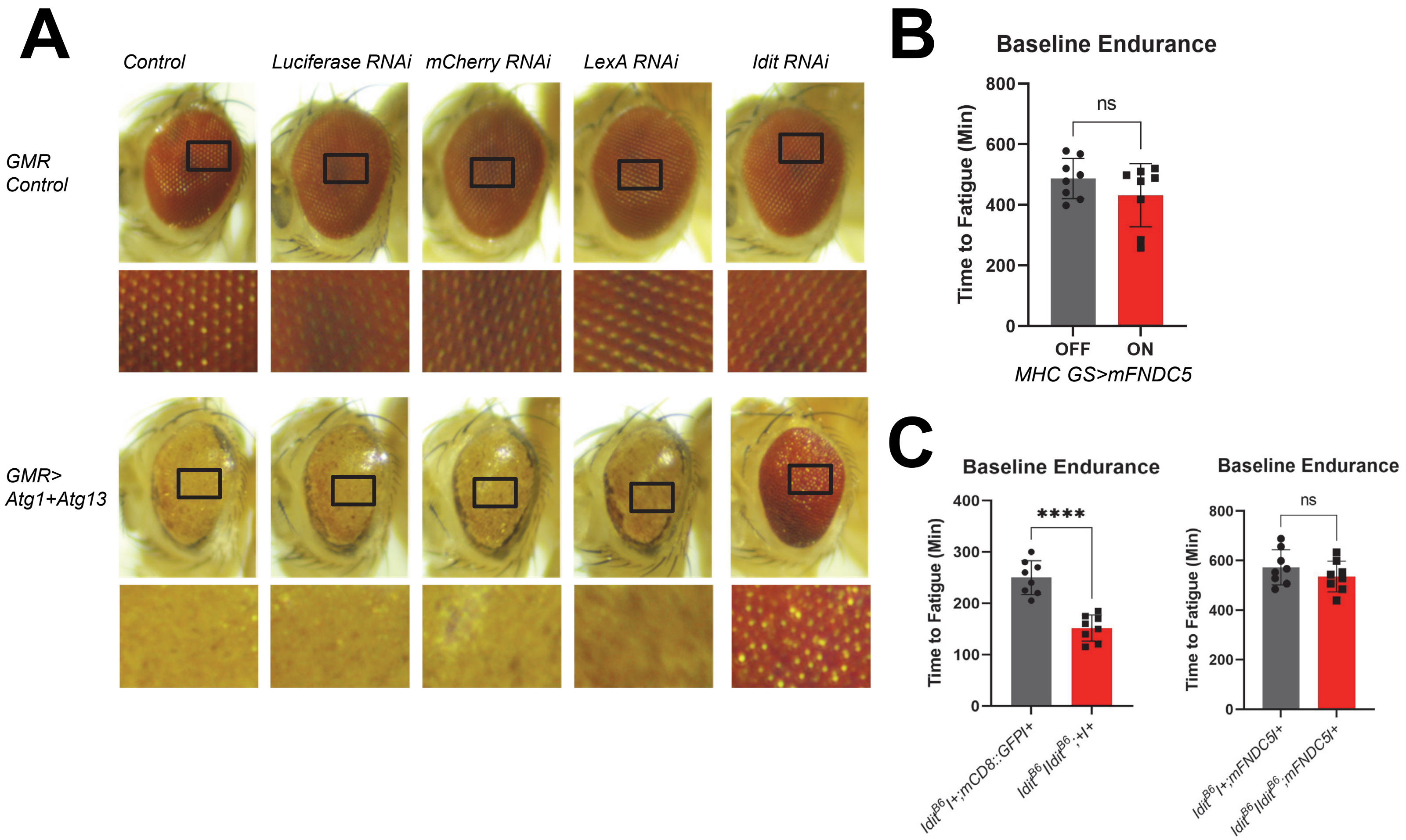

Figure S1 *mFNDC5* is phenotypically similar to *Idit*. (**A**) micrographs of adult Drosophila eyes from indicated genetic combination. Boxes are magnified below. *GMR>Atg1+Atg13* transgene produces strong eye degeneration, which is suppressed by *Idit*^{RNAi} transgenic expression. (**B**) Muscle-specific *mFNDC5* expression does not significantly extend endurance in a wild-type background (n=8, unpaired t-test, p = 0.22). (**C**) Left: *Idit*^{B6} heterozygotes (expressing *mCD8::GFP*) have higher endurance than *Idit*^{B6} homozygous mutants (n=8, unpaired t-test, p<0.0001). Right: expression of *mFNDC5* by *Idit-Gal4* rescues endurance of homozygous *Idit*^{B6} mutants to the level of unaffected *Idit*^{B6} heterozygotes (n=8, t-test, p=0.29). Asterisks indicate significance. ns=not significant. **** p<0.0001

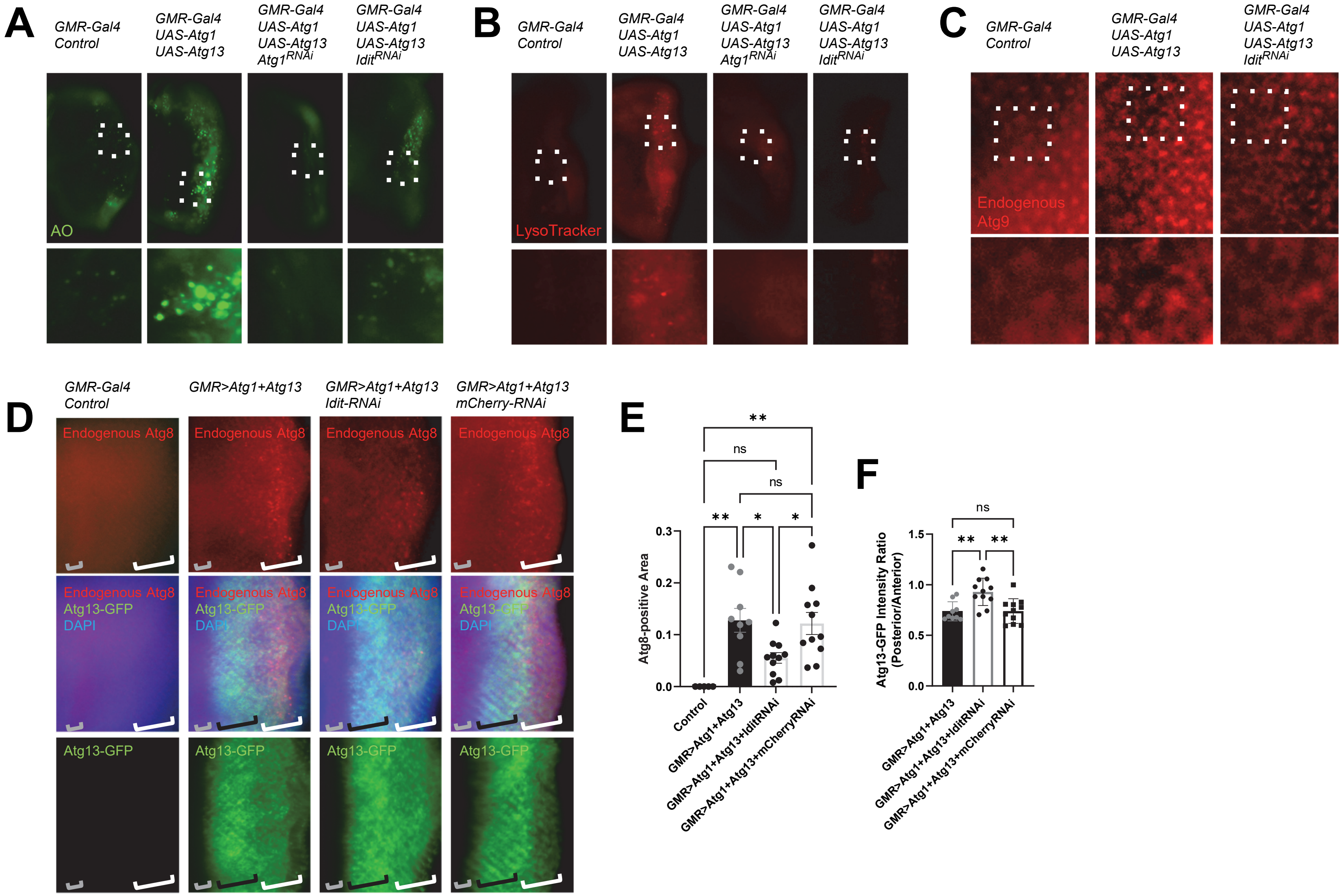

Figure S2. *Idit* silencing suppresses Atg1-Atg13-induced autophagy. (**A and B**) Developing eye discs from wandering-stage third instar larvae expressing indicated transgenes were analyzed by acridine orange (AO) staining (**A**) and Lysotracker staining (**B**) to visualize dying cells and autolysosomes, respectively. Brackets indicate the areas of differentiated ommatidia that express *GMR-Gal4*. Boxed areas are magnified in the panels below. (**C**) Developing eye discs from wandering-stage third instar larvae expressing indicated transgenes were analyzed by anti-Atg9 staining, which monitors trafficking of endosomes to autophagosomes. Boxed areas are magnified in the panels below. (**D**) Fluorescence staining of endogenous Atg8 (red) and visualization of Atg13-conjugated GFP (green) in eye discs of indicated genotypes. Grey brackets: morphogenetic furrow region, Black and white brackets: anterior/early and posterior/late areas of post-morphogenetic furrow regions, respectively. (**E and F**) Quantification of Atg8 intensity (**E**) and Atg13-GFP ratio between anterior and posterior areas of post-morphogenetic furrow region (**F**). Error bars represent standard deviation. Asterisks indicate significance from Tukey's multiple comparison test; * $p<0.05$, ** $p<0.01$.

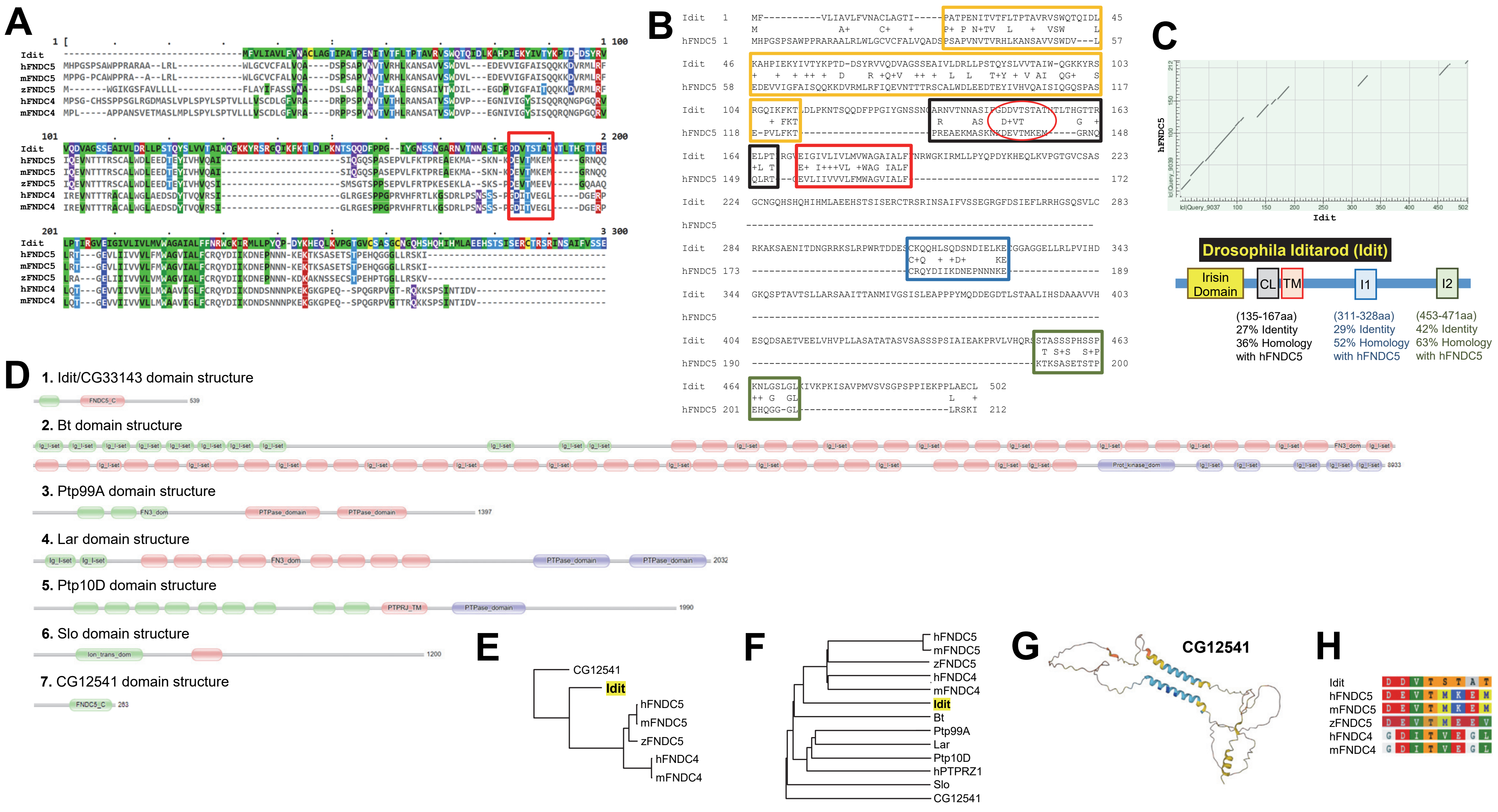

Figure S3. *Idit* is the *Drosophila* homolog of Irisin/FNDC5. (**A**) Constraint-based Multiple Alignment of Idit and its related proteins. Red box indicates the putative Irisin cleavage motif, which is further magnified in (**H**). (**B and C**) Needleman-Wunsch global alignment of Idit and human FNDC5 (**B**) and subsequent dot plot analysis (**C**) that identifies putative domains including Irisin domain (yellow boxes), Irisin cleavage domain (black boxes, CL), transmembrane domain (red boxes, TM), Intracellular domain 1 (blue boxes, I1), and Intracellular domain 2 (green boxes, I2). Homology of each domain to human FNDC5 is assessed after Needleman-Wunsch alignment of each domain. Homology information for Irisin domain and TM domain can be found in **Fig. 2B**. Putative Irisin cleavage motif is highlighted in red circle (**D**) Domain structures of Idit-related proteins in *Drosophila*. Domain structure diagrams for Idit, Bt, Ptp99A, Lar, Ptp10D, Slo, and CG12541, which are the proteins that have substantial sequence homology to human Irisin/FNDC5, were obtained from Flybase and presented here. Protein domains were identified from Pfam. CG12541 lacks the fibronectin III domain (FN3_dom) that provides the Irisin homology. All unlabeled green and red domains correspond to FN3_dom. Except Idit and CG12541, all other proteins contain unrelated domains, such as immunoglobin, protein kinase, protein phosphatase and ion channel domains. The illustrations, while not accurately to scale, are approximately representative of the intended proportions of domains. Accurate protein lengths are indicated. (**E and F**) Phylogenetic tree of Idit and related proteins, constructed through Fast Minimum Evolution (**E**) and Cobalt tree dendrogram (**F**) implemented in NCBI. Total 13 proteins (shown in **F**) were analyzed; however, in Fast Minimum Evolution analysis, 6 proteins showed sequence differences of greater than 85%, so were excluded in the analysis. (**G**) Alphafold prediction of CG12541, which revealed no structures related to the Irisin domain. (**H**) Close-up view of the putative Irisin cleavage motif.

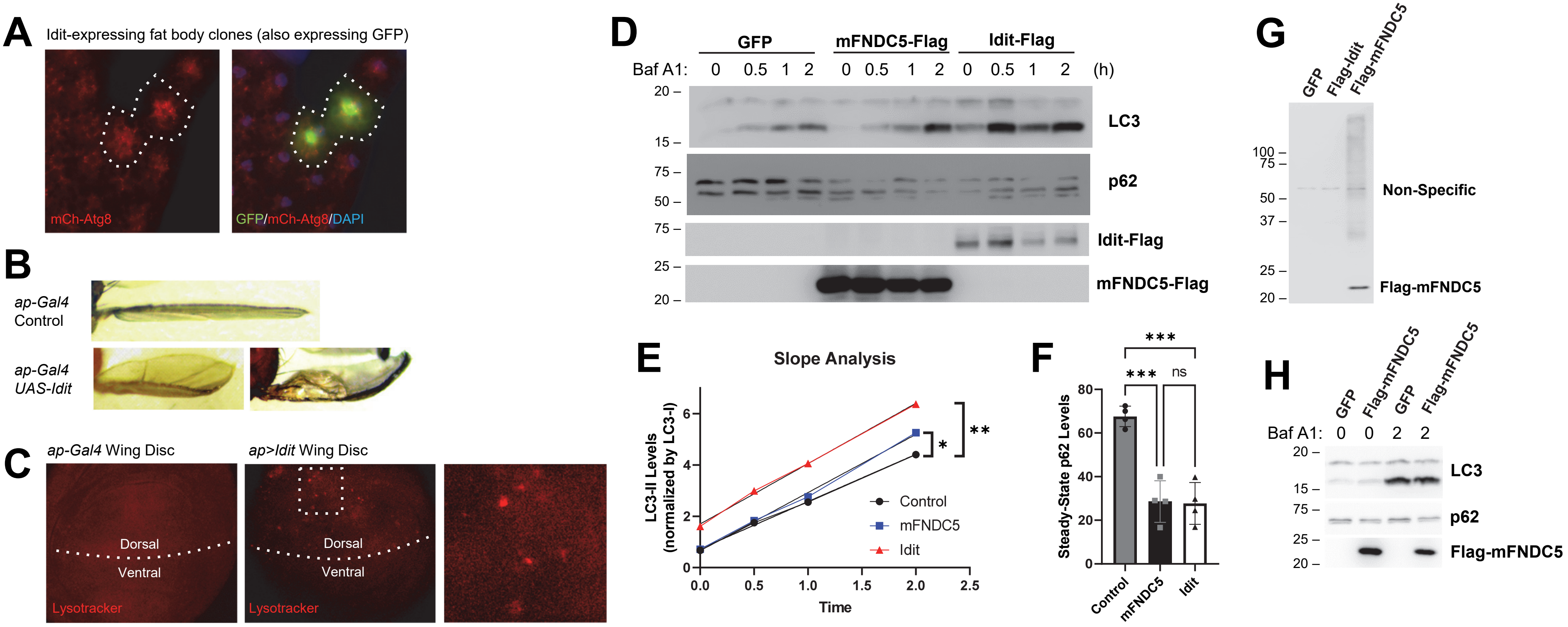

Figure S4. *Idit* overexpression produces ectopic autophagy. (**A**) Fat bodies from feeding-stage third instar larvae with somatic clones expressing both GFP and *Idit*. mCherry-labeled Atg8a was expressed throughout fat body using a fat body-specific R4 promoter. Boundaries between GFP-positive and -negative cells were marked in dotted white line. (**B**) Adult wing blades of flies expressing indicated trangenic elements. Dorsal side is up. (**C**) Developing wing imaginal discs of wandering-stage third instar larvae of indicated genotypes were stained with Lysotracker. Dorsoventral boundaries were indicated in dotted lines. Lysotracker-positive signals were only observable in dorsal compartments of *ap>Idit* wing discs. Dotted box is magnified in the right panel. (**D-H**) HEK293 cells expressing indicated constructs were treated with Bafilomycin A1 for indicated time, and then subjected to the western blot analysis of indicated proteins. In **D-F**, C-terminal Flag tag was used, while N-terminal Flag tag, which potentially disrupts signal sequence and membrane topology, was used in **G and H**. The results in (**D**) were quantified to estimate autophagic flux in LC3-II/I slope analysis (**E**) and steady state p62 level analysis (**F**). Asterisks indicate significance from linear regression analysis (**E**; * $p<0.05$, ** $p<0.01$) and Tukey's multiple comparison test (**F**; *** $p<0.001$).

Supplemental Methods

Drosophila strains and culture conditions

UAS-myc-Atg1 (*UAS-Atg1*) and *UAS-Atg13-GFP* (*UAS-Atg13*) were gifts from Dr. Thomas Neufeld (University of Minnesota). *GMR-Gal4* (#1104), *Mef2-Gal4* (#27390), *MHC-GS-Gal4* (#43641), *ap-Gal4* (#3041), *UAS-Idit^{dsRNA}* (#28823), *UAS-Atg1^{dsRNA}* (#26731), *UAS-mCD8::GFP* (#32185), *UAS-luciferase^{dsRNA}* (#31063), *UAS-mCherry^{dsRNA}* (#35785), *UAS-LexA^{dsRNA}* (#67947), *UAS-CG12541^{dsRNA}* (#64865), and *Idit^{MI03535}* (#37005) were from Bloomington Drosophila Stock Center (BDSC). *y w*, *UAS-spargel* (1, 2), and *GMR-Gal4; UAS-Atg1 UAS-Atg13/TM6B* (3) were described. *y w hsFlp; UAS-Dcr2; Act>CD2>Gal4, UAS-GFPnls, R4-mCherry-Atg8a* was a gift from Dr. Gabor Juhasz. *UAS-spargel* was a gift from Dr. David Walker. To generate *UAS-Idit* line, full-length (RE70324) Idit cDNA was obtained from Drosophila Genomics Resource Center (DGRC), 3' tagged with 3X-FLAG, and cloned into a pUAST-attB vector. To generate *UAS-mFNDC5* line, mouse FNDC5 cDNA was obtained from addgene (#35970), and cloned in the same way as for the *UAS-Idit*. The constructs were microinjected into embryos of the attP strain (#24486 from BDSC), which has a PhiC31 integrase insertion on the X chromosome and an attP landing platform on the third chromosome. The transgene insertion was identified by presence of the mini-white marker. To generate *Idit^{B6}* line, *MiMIC-to-GAL4* conversion plasmid (pBS-KS-attB1-2-GT-SA-GAL4-Hsp70pA from DGRC) was injected into *Idit^{MI03535}* embryo with the integrase helper construct. The *MiMIC-to-GAL4* conversion was initially identified by the loss of *y^+* marker, which is present in the original MiMIC construct but not in the GAL4 construct. Then, the orientation of the SA-GAL4-Hsp70pA direction was determined through PCR-based genotyping procedure. *Idit^{B6}* is one of the lines which is a proper gene-trap allele, where the GAL4 protein, instead of the Idit protein, is expressed under the endogenous *Idit* promoter. To generate the *Idit^{B6}* Rescue line, *Idit^{B6}* on the second chromosome was combined with *UAS-Idit* on the third chromosome. GeneSwitch drivers were turned on by adding 100 µm mifepristone solution to food which flies consumed three days prior to experimentation and throughout the exercise training protocol. The flies were cultured on standard cornmeal-agar medium (for strain maintenance and breeding) or 10% sugar-yeast medium (for postdevelopment husbandry, exercise training and lifespan assay) with humidity (70%), temperature (25ºC) and light (12/12 h light/dark cycle) control, unless otherwise indicated.

Antibodies, Immunoblotting and Fluorescence Imaging

Rabbit anti-Idit antibodies were made as follows: Idit coding sequence was cloned into pGEX and transformed into *E.coli* BL21 strain. Insoluble GST-Idit proteins were purified from SDS-PAGE bands and injected into rabbits (Pocono farms, Inc.). Sera were subjected to affinity purification using PVDF-immobilized proteins. Atg1 and anti-Atg9 antibodies were formerly described (3). Atg13-GFP was detected using anti-GFP antibodies (Santa Cruz Biotechnology, sc-9996; 1:50). Anti-TUBA/a-tubulin (Sigma, T5168; 1:1000), anti-ACTA1/actin (DSHB, JLA20; 1:100), anti-GABARAP (anti-Atg8a; IHC – 1:100; see (3) regarding cross-reactivity information), anti-Atp5a (Invitrogen; 1:500) antibody was used to detect endogenous levels of corresponding *Drosophila* proteins. For immunoblotting, cell and tissue lysates prepared in RIPA buffer (50 mM Tris-HCl pH 7.4; 150 mM NaCl; 1% deoxycholate Na; 1% NP-40; 0.1% SDS and complete protease inhibitor cocktail (Roche)) were boiled in 1X SDS sample buffer for

5 min at 95°C. For detection of Idit, tissue lysates were processed in 1X Lammeli buffer (1610737, Bio-Rad) at 60°C water bath for 5 min, chilled at ice for 5 min, and stored at 4°C afterwards. The processed lysates were separated by SDS-PAGE, transferred to PVDF membranes and probed with primary antibodies and then with horseradish peroxidase-conjugated secondary antibodies. Chemiluminescence was detected using LAS4000 or AI680 (GE) systems. LysoTracker Red staining, acridine orange staining and live fluorescence imaging procedures are formerly described (3). Fluorescence images were quantified in ImageJ or Adobe Photoshop as previously performed (3-5) with some modifications. Heart tube area was measured and contrast was increased to identify Atg8-positive (red) pixels above a predetermined intensity threshold. The area of pixels above this threshold was measured, added together, and then calculated as a percent of total heart tube area. Statistical analysis of % heart area was performed with 2-way ANOVA with post-hoc Tukey test for multiple comparisons.

Chill Coma

The chill coma assay was performed as previously described (6). Five vials containing approximately 5-7 flies were transferred to fresh, empty vials and immediately submerged into ice for two hours to induce a chill coma. Vials were removed and flies were individually monitored for recovery, which was scored when an individual fly was standing upright. Unpaired t-test or one-way ANOVA was performed to measure the statistical difference between means.

Cardiac Pacing

Cardiac pacing was performed as previously described (5). Electrodes from a square wave stimulator were placed on each end of a modified microscope slide and secured with tinfoil. Conductive gel was placed on the foil on each end, leaving a 2-3 mm gap between the gel. Flies were placed between the gel with the head touching one side of the gel and the abdomen touching the other. Hearts were paced at 40V and 6 Hz for 30 seconds. Hearts were then visually scored for failure (i.e. not beating) immediately after pacing and results were presented as failure rate per total flies paced. Data was analyzed with a Chi-square test for significance.

Mammalian Cell Culture

Idit and mFNDC5 cDNA, obtained as described above, were subcloned into a pLU-CMV vector after being tagged with N-terminal or C-terminal Flag sequences. HEK293 cells (the 293A substrain from Invitrogen, tested negative for mycoplasma by PCR) were cultured in Dulbecco's modified Eagle's medium (DMEM, Invitrogen) containing 10% fetal bovine serum (FBS, Sigma) and penicillin/streptomycin (Thermo Fisher Scientific) at 37°C in 5% CO_2. For transient expression of proteins, HEK293 cells were transfected with purified plasmid constructs and polyethylenimine (PEI, Sigma). Cells were subjected to autophagic flux assays using Bafilomycin A_1 (Sigma), as indicated in the figures, at 24 hr after transfection.

Quantitative RT-PCR

RNA was extracted from whole flies using TRIzol Reagent (Invitrogen) and was equalized to 30 ng/ul between groups. Relative mRNA abundance of *Idit* was measured with one-step RT-PCR. Reactions containing template RNA, SYBR Green (Applied Biosystems), MultiScribe reverse transcriptase (Applied Biosystems), RiboLock Rnase inhibitor (Thermo Scientific), primers, and water were run in a QuantStudio 3 RT-PCR System (Applied Biosystem). Comparative Ct (7) was used to quantitate relative abundance and fold change of *Idit* mRNA using *Act5c* as an internal control. Unpaired t-test was used to test for significance. Primers used are as follows:

Act5c Forward: CGCAGAGCAAGCGTGGTA
Act5c Reverse: GTGCCACACGCAGCTCAT

Idit Forward: AGCAACGACATCGAGCTGAA
Idit Reverse: CGCTAGCAGACTAGTCACGG

References to Supplemental Methods

1. M. J. Tinkerhess *et al.*, The Drosophila PGC-1alpha homolog spargel modulates the physiological effects of endurance exercise. *PLoS One* **7**, e31633 (2012).
2. S. K. Tiefenbock, C. Baltzer, N. A. Egli, C. Frei, The Drosophila PGC-1 homologue Spargel coordinates mitochondrial activity to insulin signalling. *EMBO J* **29**, 171-183 (2010).
3. M. Kim *et al.*, Drosophila Gyf/GRB10 interacting GYF protein is an autophagy regulator that controls neuron and muscle homeostasis. *Autophagy* **11**, 1358-1372 (2015).
4. D. Damschroder, T. Cobb, A. Sujkowski, R. Wessells, Drosophila Endurance Training and Assessment of Its Effects on Systemic Adaptations. *Bio Protoc* **8**, e3037 (2018).
5. R. J. Wessells, E. Fitzgerald, J. R. Cypser, M. Tatar, R. Bodmer, Insulin regulation of heart function in aging fruit flies. *Nat Genet* **36**, 1275-1281 (2004).
6. T. Cobb, D. Damschroder, R. Wessells, Sestrin regulates acute chill coma recovery in Drosophila melanogaster. *Insect Biochem Mol Biol* **133**, 103548 (2021).
7. T. D. Schmittgen, K. J. Livak, Analyzing real-time PCR data by the comparative C(T) method. *Nat Protoc* **3**, 1101-1108 (2008).
